# Green preparation and evaluation of bentonite supported Bi_2_O_3_/polyester nanocomposites as gamma radiation shielding materials

**DOI:** 10.1038/s41598-026-53392-9

**Published:** 2026-06-09

**Authors:** Mohamed A. Hamada, Olfat M. Sadek, W. K. Mekhamer, Shoaa M. Al-Balawi, M. Elsafi, Ibrahim H. Saleh

**Affiliations:** 1 Materials Science Department, Institute of Graduate Studies and Research, Alexandria University, Alexandria, Egypt; 2 Department of General Science Program, College of Deanship of Support Studies, Alasala Colleges, Dammam, Saudi Arabia; 3 Physics Department, Faculty of Science, Alexandria University, Alexandria, 21511 Egypt; 4 Environmental Studies Department, Institute of Graduate Studies and Research, Alexandria University, Alexandria, Egypt

**Keywords:** Bi_2_O_3_-NPs, Bentonite, Olive leaves, Unsaturated polyester, Nanocomposites, Radiation shielding, Chemistry, Materials science, Nanoscience and technology

## Abstract

In this work, a novel mixture of Bi_2_O_3_-NPs and bentonite was prepared using green synthesis method using olive leaves extract where Bi_2_O_3_-NPs were formed and loaded on the surface of bentonite layers to get homogeneous mixing between Bi_2_O_3_-NPs and bentonite. The successful preparation of this mixture was confirmed using TEM, IR and XRD. After that, unsaturated polyester nanocomposites with varying ratios (1, 5, 10, and 20 wt%) were prepared using this synthesized mixture. These composites were evaluated as shielding materials against gamma radiation, since different shielding parameters were measured at different energies (59, 661, (1173 and 1333 keV) from Am-241, Cs-137, and Co-60, respectively. Also, the thermal and mechanical properties were measured, where the 20 wt% sample only exhibits 50% weight loss after 402 °C, as opposed to 357 °C for pure unsaturated polyester. On the other hand, results of compressive strength showed slight enhancement with addition of bentonite compared to only Bi_2_O_3_ nanocomposites. The results showed the improvement of shielding properties with increase of filler ratio, where the 20 wt% nanocomposite sample has highest LAC values among all samples where it has LAC values of 1.133, 0.126, 0.091, and 0.090 cm^-1^ at 59, 661, 1173, and 1333 keV, respectively. This demonstrates the importance of using these nanocomposites as radiation shields against low-energy photons, since it can achieve a shielding efficiency of 90% at 59keV.

## Introduction

Nuclear power plants, industry, medicine, and scientific research are just a few of the fields that use gamma and X-rays^1–3^. However, both people and the ecosystem suffer when ionizing radiation exposure occurs unintentionally. It could lead to changes in cells, harm to organs, and other health problems^4,5^.

Controlling the time of exposure, distance from the radiation source, and the use of shields can all help reduce the amount of radiation that is absorbed^3,6^. High atomic number micro and nano metal oxide reinforcement improves the radiation attenuation capabilities of polymers and qualifies them for usage as shields^2,5,7^. In addition, compared to other materials used for shielding, polymer composites are lightweight, flexible, and mechanically strong^7–9^.

Bismuth oxide (Bi_2_O_3_) at the nanoscale is one of the metal oxides that is utilized. For bismuth oxide, bismuth has a higher atomic number (Z = 83) and bismuth oxide also has a high density. Unlike lead, it is also non-toxic^10^. To create polymer composites for shielding applications, numerous researchers employed bismuth oxide nanoparticles. El Sharkawy et al. investigated the shielding capabilities of recycled PVC doped with varying proportions of Bi_2_O_3_ nanoparticles at varying energy released from the Eu-152 source^10^. Additionally, the shielding capabilities of HDPE composite reinforced with Bi_2_O_3_ NPs were investigated by Abdalsalam et al.^11^. Elsafi et al. used Bi_2_O_3_ NPs to create epoxy composites for shielding applications^12^. Also, bismuth oxide was added to PMMA by Cao et al. to improve its shielding qualities, and subsequent investigations combined bismuth oxide with various polymers^5,7,13^.

A form of thermoset polymer, unsaturated polyester finds employment in a wide range of industries, including building, automotive, military goods, aerospace, and the maritime sector. It is distinguished by its good mechanical qualities, inexpensive cost, and ease of processing^4,14,15^. Unsaturated polyester was employed by numerous researchers as a matrix in composites for radiation shielding applications. Bagheri et al. evaluated the shielding properties of unsaturated polyester after adding varying amounts of PbO together with a small amount of nano clay^4^. More et al. investigated the attenuation characteristics of an unsaturated polyester composite loaded with TiO_2_ NPs against gamma rays and neutrons^16^. For gamma shielding applications, Hemily et al. created unsaturated polyester composites reinforced with WO_3_ NPs and leftover marble^17^. Additionally, more et al. investigated the shielding qualities of unsaturated polyester after adding SnO_2_ NPs^9^. Unsaturated polyester composites for shielding applications were also the subject of several research studies^1,18–20^.

Although importance of Bi_2_O_3_ NPs in improvement of unsaturated polyester’s shielding properties, it negatively affects mechanical properties of unsaturated polyester. The previous work showed a decrease in compressive strength with increase of Bi_2_O_3_ NPs addition percentage^21^. Also, other studies reported the effect of inorganic nanoparticles on mechanical properties of unsaturated polyester. Bagheri et al. found that the tensile strength of unsaturated polyester composites decreases with increasing PbO amount^4^. Gamea et al. reported the decreasing of tensile strength with increasing of electric furnace dust ratio above 15 wt%^22^. Baskaran et al. observed that tensile strength starts to decrease with increasing of CaCO_3_ nanoparticles percent above 5 wt% in unsaturated polyester composites^23^. Also, Baskaran et al. reported the same behavior for nano alumina filled unsaturated polyester composites^24^. Shoaib et al. showed that optimized addition percentage of TiO_2_ NPs in unsaturated polyester is 4 wt% and the tensile strength starts to decrease above it^25^.

Bentonite is one of the most materials used in reinforcement of polymeric materials due to its good mechanical and chemical properties in addition to it is ecofriendly and cheap^26,27^. The addition of bentonite to unsaturated polyester can improve its thermal and mechanical properties^28^. Many researchers used clays in fabrication of composites for shielding applications. Mahmoud et al. added halloysite clay to epoxy with different percent and reported increasing of linear attenuation coefficient with increasing of clay percent from 0 to 40 wt%^29^. Also, Bagheri et al. used 5 wt% of nano clay with different wt% of PbO in fabrication of unsaturated polyester composites and they stated that addition of nano clay enhanced tensile strength of unsaturated polyester^4^. On the other hand, elsafi et al. added different wt% mixture from red clay and Bi_2_O_3_ NPs to epoxy resin^30^. Also, El Sharkawy et al. prepared low-cost radiation shielding nanocomposite based on polypropylene containing different amounts of CdO NPs and bentonite^31^. But they didn’t mention much more about the effect of clays on mechanical properties of shielding polymer composites. So, we focused in this work on moderation of previously mentioned negative effect of Bi_2_O_3_ on compressive strength of unsaturated polyester composites.

The noticed difference between densities of bentonite and Bi_2_O_3_ may lead to inhomogeneous mixture during fabrication of unsaturated polyester composites. To achieve good homogeneity between Bi_2_O_3_ and bentonite, Bi_2_O_3_ NPs can be synthesized in presence of bentonite where it is formed and loaded at surface of bentonite. Different researchers prepared this combination using chemical method^32–34^. On the other hand, green synthesis is reported as cost effective, non-toxic and successful method to load metal oxide nanoparticles on bentonite surface. Golmohammadi et al. synthesized ZnO NPs supported on bentonite using extract of jujube fruit^35^. Also, Aaga et al. used croton macrostachyus leaves extract for synthesis of ZnO / bentonite nanocomposite^36^. In addition, Choudhary et al. prepared Ag NPs on montmorillonite clay using extract of sida acuta plant leaves^37^.

In the current study, olive leaves extract was used to prepare Bi_2_O_3_ NPs supported on bentonite utilizing the green synthesis approach where it is rich with various phytochemicals like polyphenols, flavonoids, and terpenoids which act as reducing agent for metal ions and stabilizing agent for produced nanoparticles. After that, these nanoparticles were used in fabrication of unsaturated polyester nanocomposites with different filler loading. The attenuation properties of these nanocomposites were studied at different energies as well as the effect of bentonite addition on its thermal and mechanical properties.

## Materials and methods

### Materials

Olive leaves were bought from local market. Bismuth nitrate pentahydrate Bi(NO_3_)_3_ 0.5H_2_O were supplied by universal fine chemicals Ltd. Glacial acetic acid CH_3_COOH was obtained from fisher scientific company. Sodium bentonite was provided from elnasr mining company. Pre-accelerated unsaturated polyester resin was supplied from SUPIC Co. Methyl ethyl ketone peroxide (MEKP) was obtained from AKPA chemicals.

### Preparation of olive leaves extract

Olive leaves were first sliced into little bits. After that, 40 g of chopped leaves were heated for 60 min at 95 °C in 500 ml of distilled water. The extract was then allowed to cool in the air, filtered, and refrigerated for later use.

### Green synthesis of bentonite supported Bi_2_O_3_ NPs

**Fig. 1:**
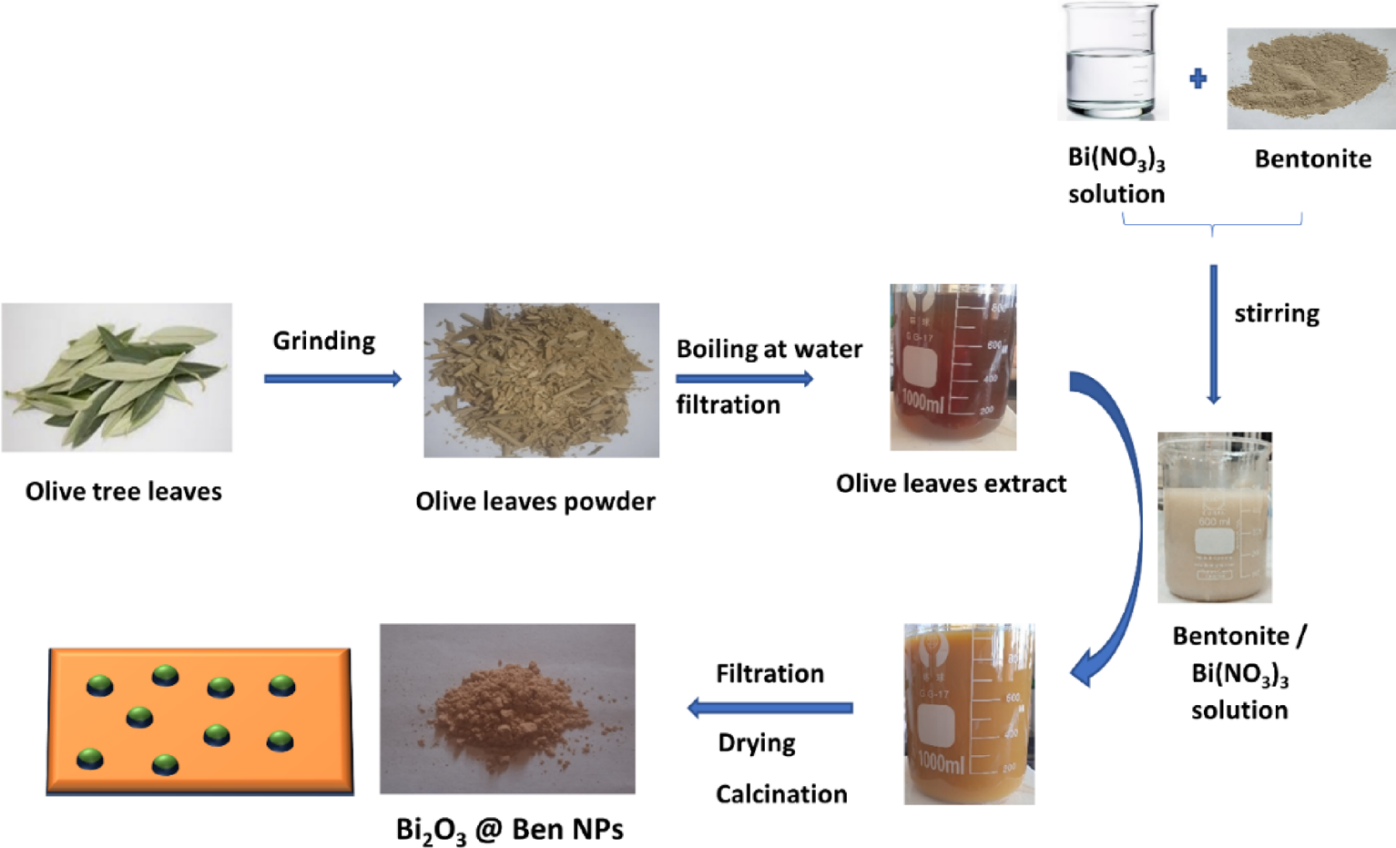
Green synthesis of bentonite supported Bi_2_O_3_ NPs.

Initially, 50 ml of 0.1 M Bi(NO_3_)_3_ solution was prepared by dissolution of 2.425 gm Bi(NO_3_)_3_.5H_2_O in 5 ml of acetic acid at 60 °C. Then, it was completed to 50 ml with distilled water. 1 gm of bentonite was stirred with 50 ml of this Bi(NO_3_)_3_ solution until good dispersion was achieved. Then, 400 ml of olive leaves extract was added dropwise to this suspension at 60 °C under vigorous stirring. The mixture was stirred for 3 h under the same conditions. After this period, the resultant precipitate was filtered, washed several times with distilled water, then dried in an oven at 70 °C for 4 h and subsequently calcinated at 600 °C for 2 h. Figure 1 summarizes steps of nanoparticles synthesis.

### Preparation of unsaturated polyester nanocomposites

**Fig. 2:**
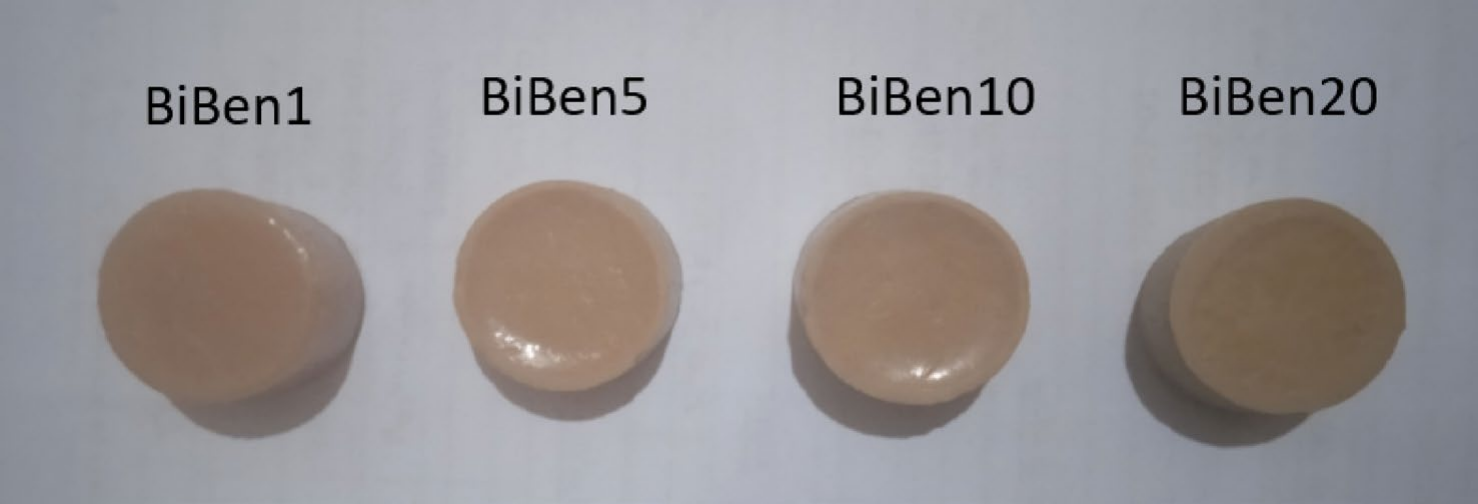
image of different composites samples.

Unsaturated polyester resin was blended with the prepared nanoparticles of varying weight percentages (0, 1, 5, 10, and 20) and then sonicated for 30 min. After that, the mixture was stirred for half an hour. The curing reaction was then started by adding 1% of MEKP to the mixture. The mixture was then allowed to solidify after being put into cylindrical molds with diameter of 2.2 cm and different thickness (0.5, 1, and 1.5 cm). The composite samples, along with their density and composition, are displayed in Table 1 and Fig. 2.

**Table 1:** composition and density of composite samples.

	Composition (%)		Density (gm/cm^3^)
	Unsaturated Polyester	Bentonite supported Bi_2_O_3_ NPs	
UP	100	0	1.306 ± 0.03
BiBen1	99	1	1.302 ± 0.03
BiBen5	95	5	1.346 ± 0.01
BiBen10	90	10	1.416 ± 0.04
BiBen20	80	20	1.502 ± 0.04

### Characterization

The size and shape of the prepared nanoparticles was measured by transmission electron microscope (JEOL JEM-1400 plus). IR spectra of the nanoparticles and nanocomposites were measured by a PerkinElmer spectrum two FT-IR spectrometer in the wave number range of 4000–450 cm^-1^. XRD patterns were measured by a Bruker XRD D2 phaser diffractometer using Cu Kα radiation (λ = 1.54 Å). Thermal stability of nanocomposites was evaluated with heating rate 10 C^°^/min using Q600 thermogravimetric analyzer, TA instruments. Scanning electron microscope (JEOL JSM IT200) was utilized to study fracture surface of nanocomposites and to make EDX analysis for the prepared nanoparticles. In addition, compression test was conducted for nanocomposite samples using universal testing machine (5ST, Tinius Olsen).

### Measurement of attenuation properties

**Fig. 3:**
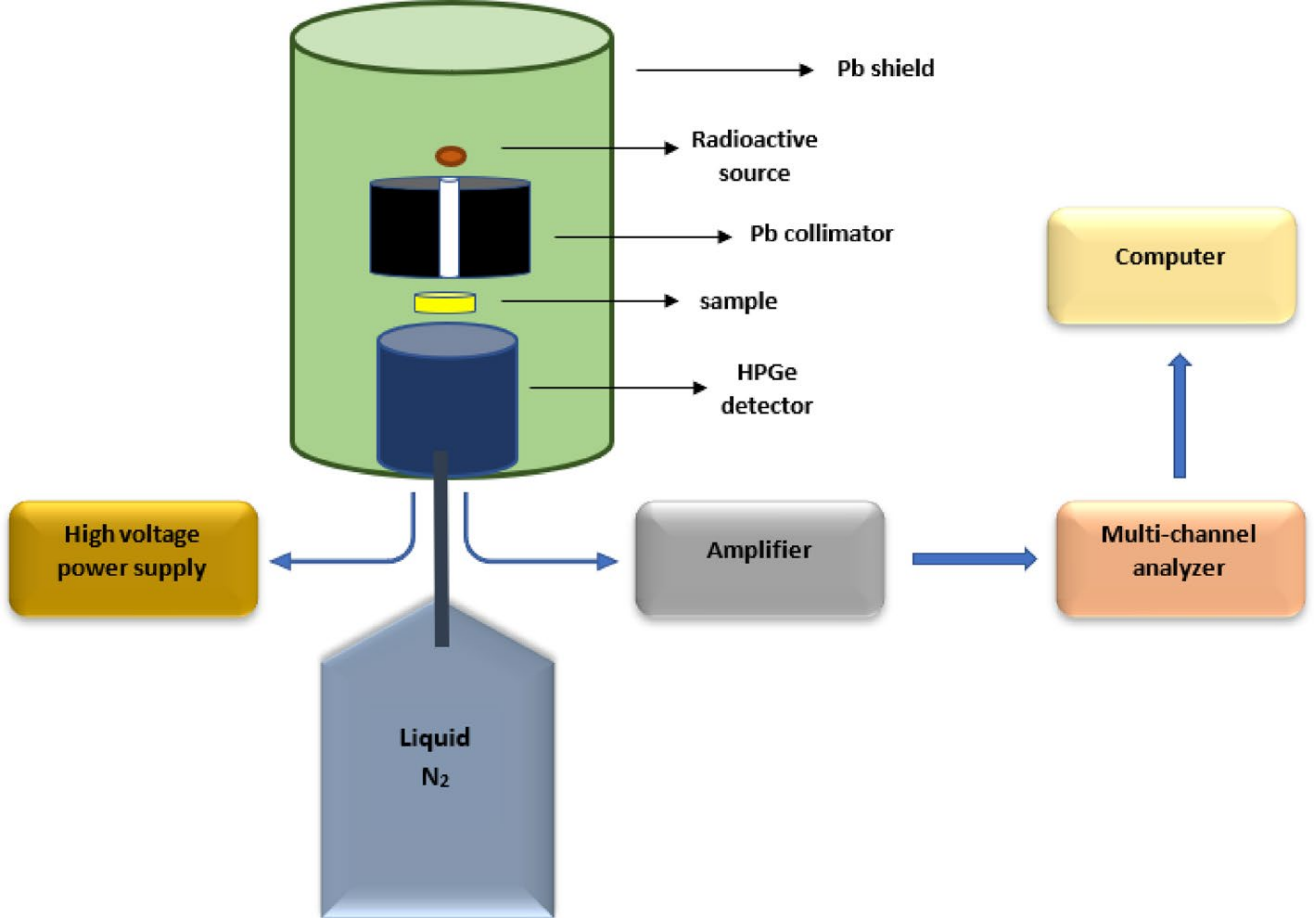
The experimental setup for measurement of attenuation coefficient.

**Fig. 4:**
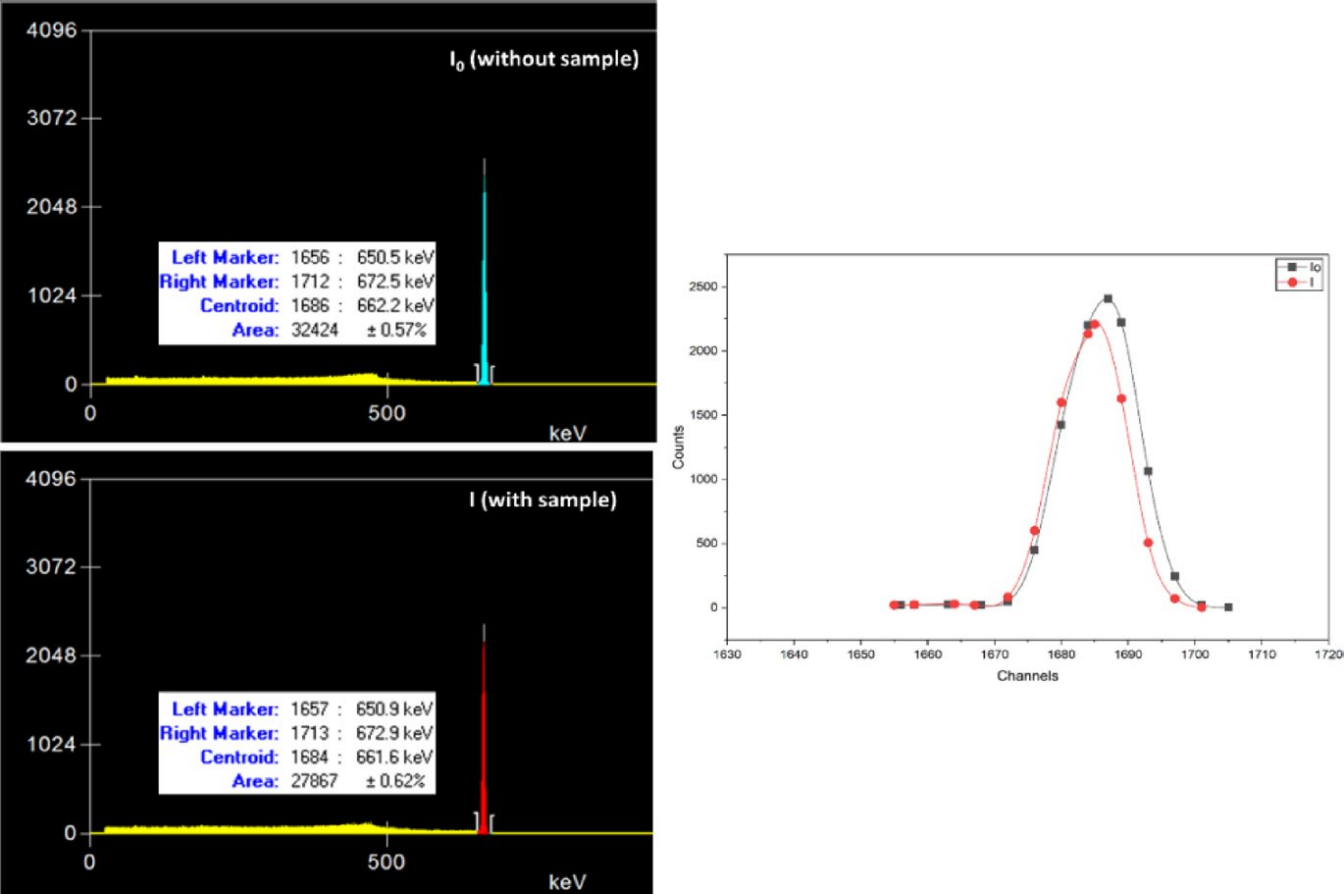
The spectra of Cs-137 in absence and presence of sample.

The attenuation characteristics of the composites were examined using a high purity Germanium (HPGe) detector and several radioactive sources emit gamma rays with different energies (Am-241 emits at 59 keV, Cs-137 emits at 661 keV, and Co-60 emits at 1173 and 1333 keV) with initial activity of 40.3 kBq in 1998. The composites samples were placed between the detector and the radioactive source as shown in Fig. 3. The peak intensity was measured by peak area at dead time ratio of 0.5% in absence and presence of the samples as shown in Fig. 4 and the linear attenuation coefficient (LAC) was determined at each energy according to the equation below:

$$\mu = \frac{1}{x} \mathrm{l}\mathrm{n}(\frac{\mathrm{I}\mathrm{o}}{\mathrm{I}})$$

where µ, I_o_, I, and x represent linear attenuation coefficient, Peak intensity in absence of sample, which represent the photopeak area (25,055, 32,424, 9872, and 9080 for Am-241, Cs-137, and Co-60, respectively), Peak intensity in presence of sample, and thickness of sample, respectively. From LAC values, other parameters of shielding such as half value layer (HVL), tenth value layer (TVL), and radiation shielding efficiency (RSE%) were measured using the following equations.

$$HVL = \frac{\mathrm{ln}(2)}{\mu }$$

$$TVL = \frac{\mathrm{ln}(10)}{\mu }$$

$$RSE\% = (1 - \frac{\mathrm{I}}{\mathrm{I}\mathrm{o}}) * 100$$

Also, theoretical linear attenuation coefficient was calculated using XCOM software by entering the chemical composition of materials where the resultant mass attenuation coefficient from XCOM software was multiplied by density of composites to obtain the theoretical linear attenuation coefficient^38^. The chemical composition of bentonite supported Bi_2_O_3_ NPs obtained from EDX analysis was used in theoretical calculations to ensure accuracy of results.

## Results and discussion

### TEM and EDX analysis of synthesized nanoparticles

**Fig. 5:**
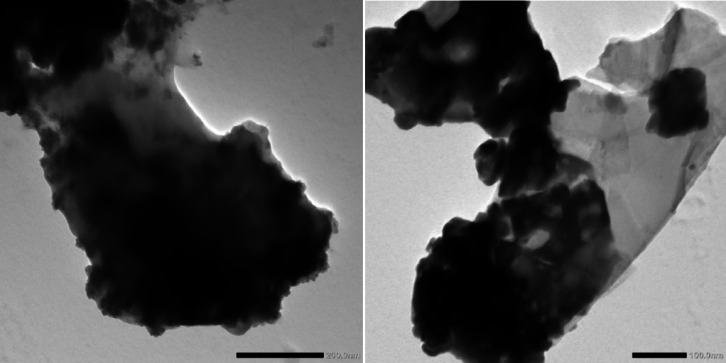
TEM images of bentonite supported Bi_2_O_3_ NPs.

**Fig. 6:**
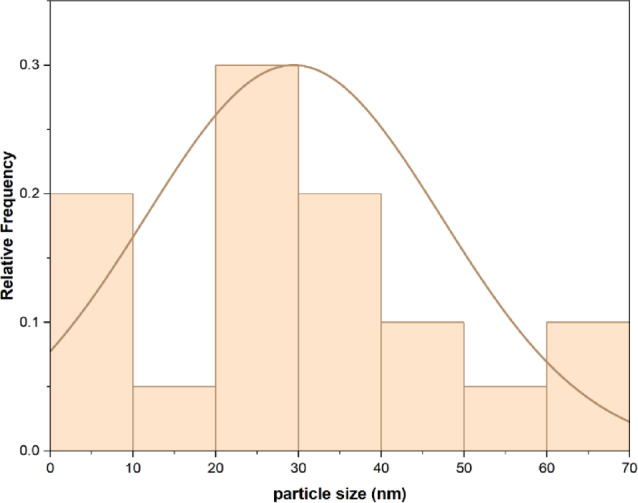
size distribution of prepared nanoparticles.

**Fig. 7:**
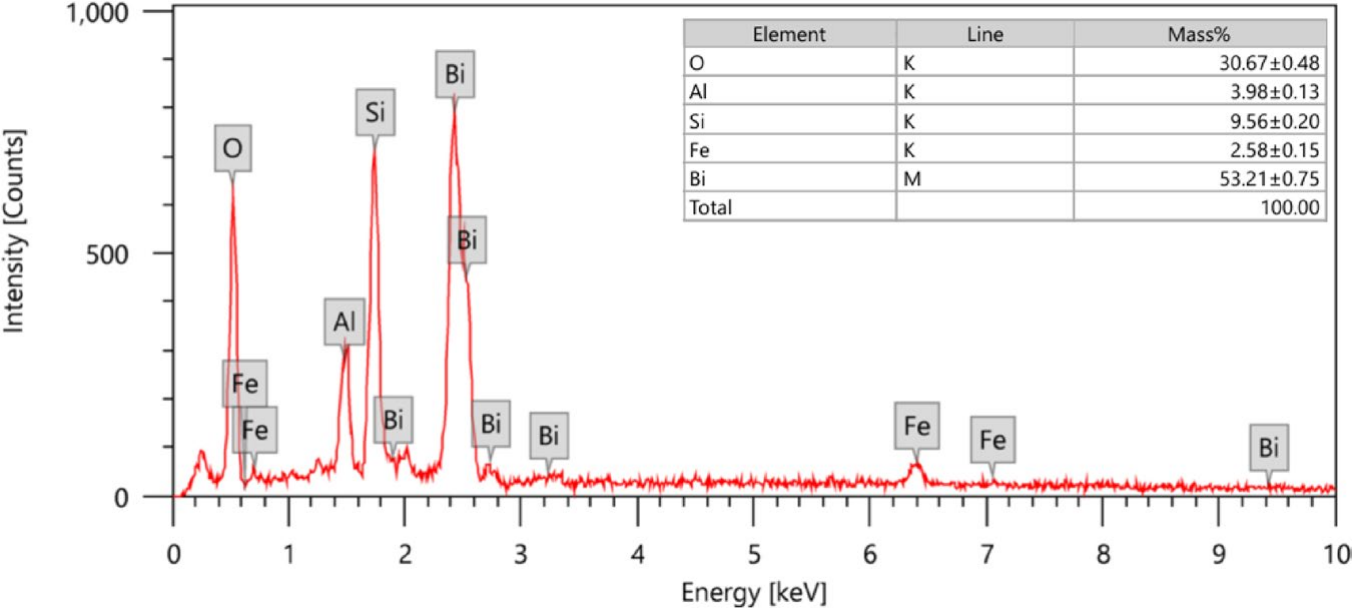
EDX spectrum of bentonite supported Bi_2_O_3_ NPs.

According to TEM images (Fig. 5), it is noticed that the dispersion of dark particles which represent Bi_2_O_3_ on the surface of lighter bentonite sheets with presence of some agglomerations. It confirms successful loading of Bi_2_O_3_ NPs on bentonite surface. Also, it is seen in Fig. 6 that the formed Bi_2_O_3_ particles are in nano size where the average size 29.4 ± 17.86 nm. In addition, the formation of Bi_2_O_3_ NPs on bentonite surface layers is confirmed by EDX analysis (Fig. 7) where it shows the presence of silicon and aluminum elements that represent main components of bentonite in addition to presence of bismuth element as major element.

### FT-IR spectroscopy

**Fig. 8:**
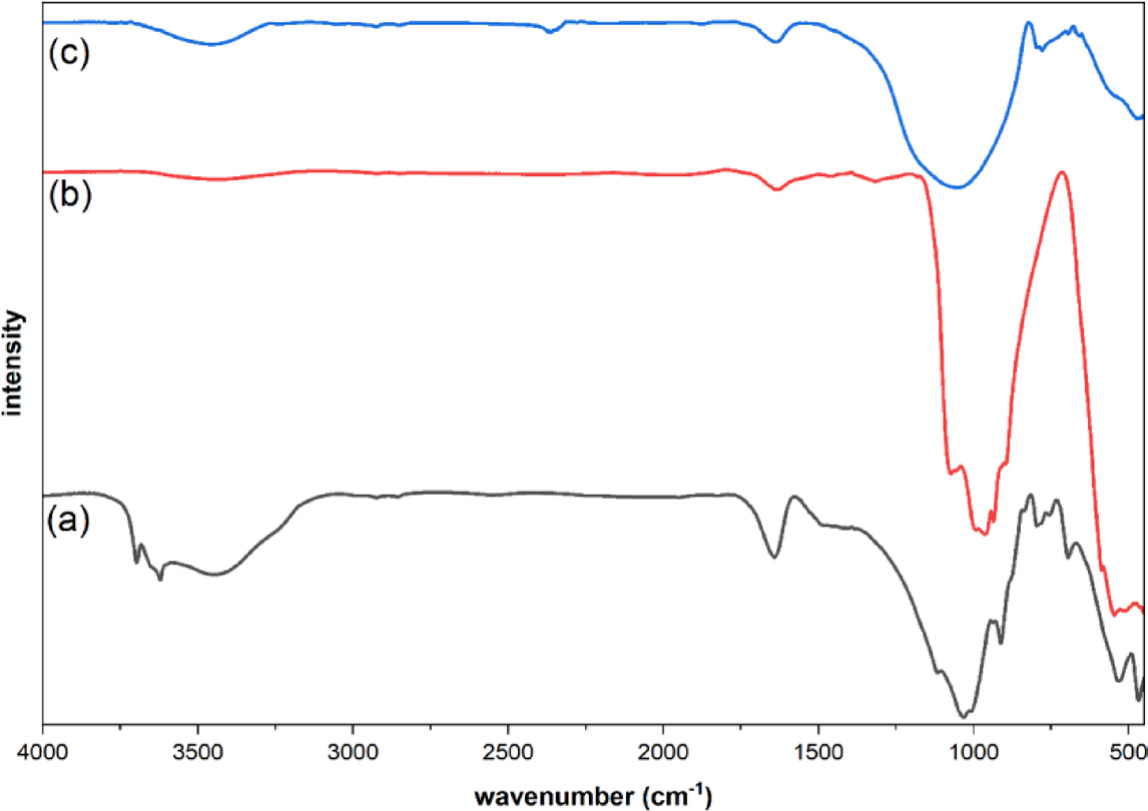
FT-IR spectra of (**a**) sodium bentonite, (**b**) Bi_2_O_3_ NPs, and (**c**) bentonite supported Bi_2_O_3_ NPs.

**Fig. 9:**
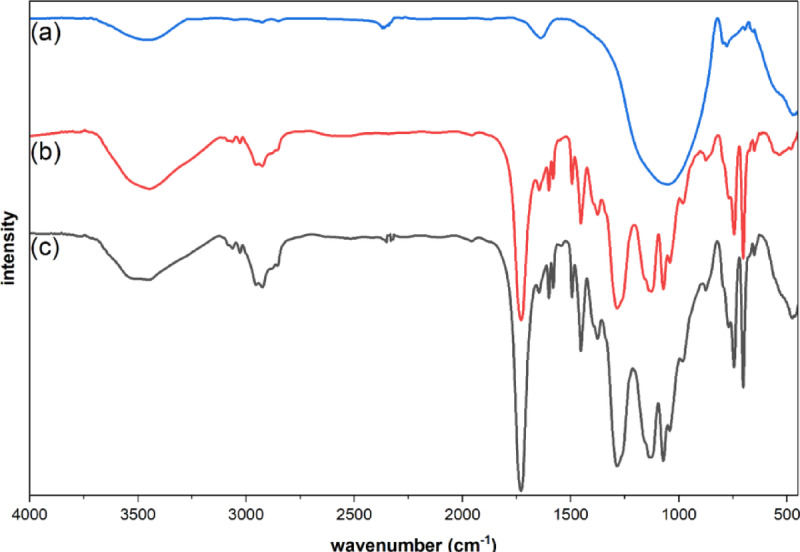
FT-IR spectra of (**a**) bentonite supported Bi_2_O_3_ NPs, (**b**) pure UP, and (**c**) BiBen20 nanocomposite.

Figures 8 and 9 show IR spectra of sodium bentonite, Bi_2_O_3_ NPs, bentonite supported Bi_2_O_3_ NPs, pure UP, and 20 wt% nanocomposite (BiBen20). As seen in IR spectrum of bentonite (Fig. 8a), the peaks at 3697 and 3623 cm^-1^ are related to structural O–H stretching^35,39,40^. The peaks at 3442 and 1642 cm^-1^ corresponding to O–H stretching and bending in adsorbed water^34,37^. Also, the peaks at 1032 and 913 cm^-1^ are attributed to Si–O and Al–O vibrations^41,42^. The peaks at 795 and 694 cm^-1^ are related to Si–O-Si vibrations and the peaks at 531 and 466 cm^-1^ corresponding to Si–O–Al and Si–O–Si bending vibrations^35,41–43^. In Fig. 8b, the peaks at 546, 937, 963, and 1074 cm^-1^ are related to Bi–O bond vibrations^44–47^. As seen in Fig. 8c, the peaks at 3461 and 1640 cm^-1^ are assigned to adsorbed water and the peak at 2361 cm^-1^ is related to adsorbed CO_2_ molecules. Also, it is noticed the appearance of broad peak at 1057 cm^-1^ which is due to overlapping between peaks of bentonite and Bi_2_O_3_ NPs. Also, disappearance of peak at 3623 cm^-1^ is attributed to removal of structural O–H through calcination process. Also, the presence of peaks at 795 and 695 cm^-1^ which are related to bentonite and the peaks at 778 and 473 cm^-1^ which are related to Bi_2_O_3_ NPs indicates the formation and loading of Bi_2_O_3_ NPs on bentonite surface. In both Fig. 9b and c, the peak at about 3460 cm^-1^ is related to O–H stretching vibration, the peak at 3028 cm^-1^ is attributed to aromatic C–H bond vibration, and the peak at 2954 cm^-1^ is related to aliphatic C–H bond stretching. While the peaks at 1728 and 1453 cm^-1^ are related to C=O bond stretching vibration and C–H bond bending vibration, respectively. The peaks at 1283, 1130, and 1072 cm^-1^ are related to C–O bond vibrations and the observed peaks at 745 and 702 cm^-1^ are related to aromatic C–H bending vibrations^48,49^. Moreover, the spectrum of 20 wt% nanocomposite shows additional peak at 476 cm^-1^ which confirms incorporation of prepared nanoparticles to unsaturated polyester. It is worth noting that no significant shift is observed in the characteristic peaks of unsaturated polyester after addition of the nanoparticles which indicates that interaction between components of composites is mainly physical.

### X-ray Diffraction

**Fig. 10:**
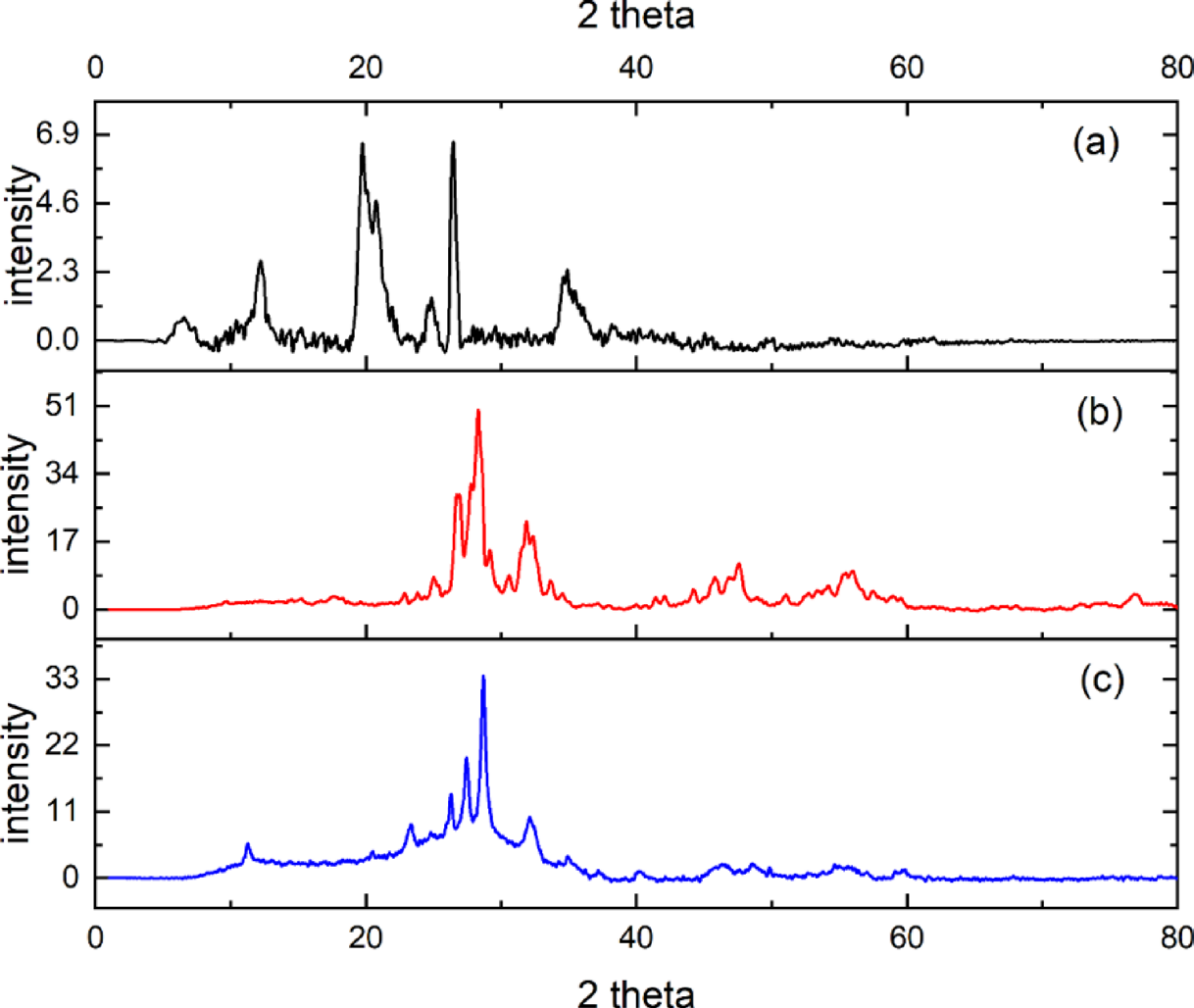
XRD patterns of (**a**) sodium bentonite, (**b**) Bi_2_O_3_ NPs, and (**c**) bentonite supported Bi_2_O_3_ NPs.

**Fig. 11:**
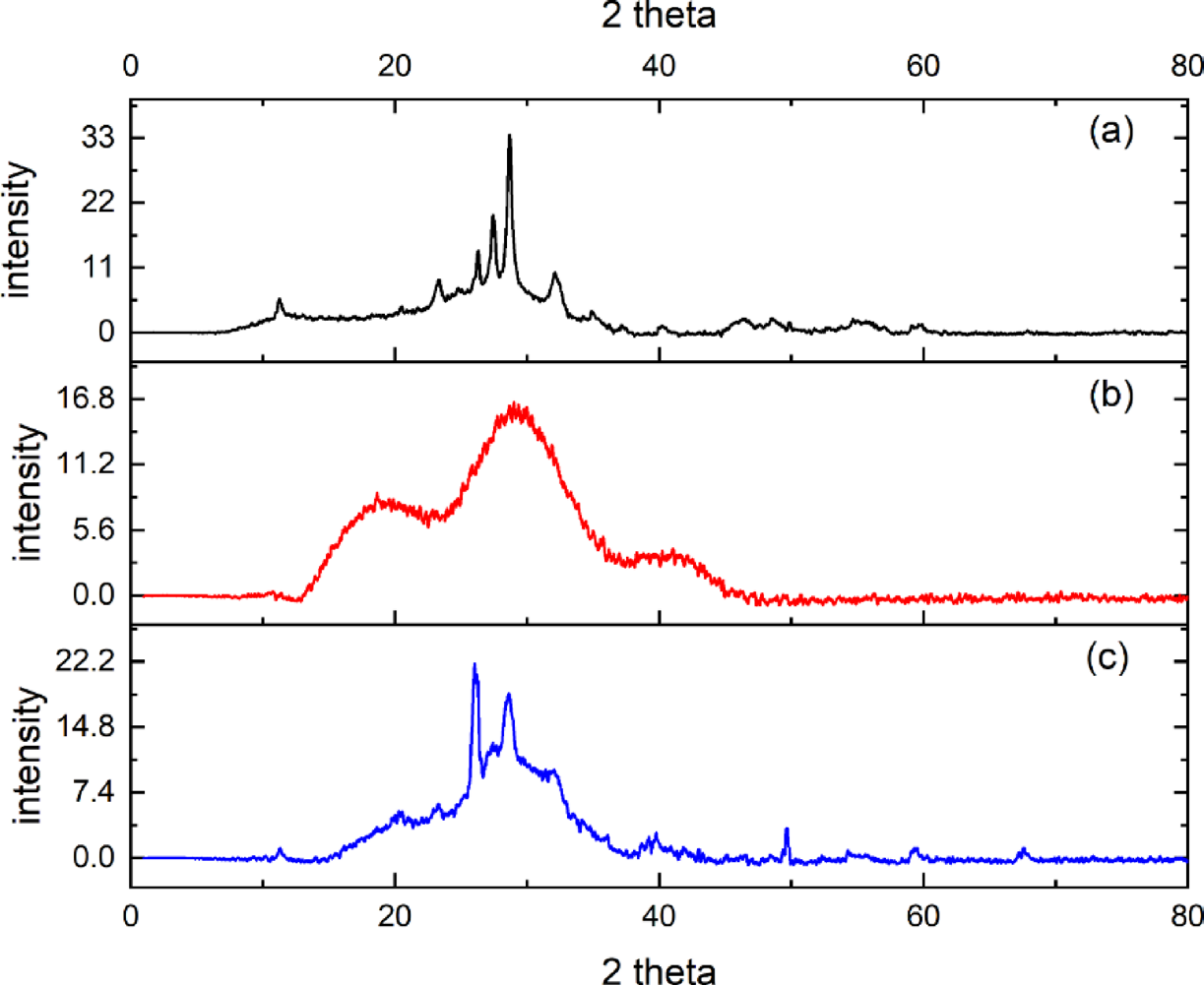
XRD patterns of (**a**) bentonite supported Bi_2_O_3_ NPs, (**b**) pure UP, and (**c**) BiBen20 nanocomposite.

Figures 10 and 11 show XRD patterns of sodium bentonite, Bi_2_O_3_ NPs, bentonite supported Bi_2_O_3_ NPs, pure UP, and 20 wt% nanocomposite (BiBen20). In Fig. 10a, the peaks at 6.5°, 19.74°, and 34.87° corresponding to montmorillonite mineral and the peak at 26.43° is related to quartz ^35^. On the other hand, the peaks at 12.19° and 24.85° may be related to kaolinite mineral ^50^. The XRD pattern of Bi_2_O_3_ NPs (Fig. 10b) shows main peaks at 26.89°, 27.76°, 28.28°, 31.84°, 32.30°^44,47,51,52^. in Fig. 10c, it is noticed the appearance of the peaks at 27.42°, 28.64°, and 32.10° which are related to Bi_2_O_3_ NPs. Also, appearance of the peaks at 11.25°, 23.30°, and 26.29° that are related to bentonite components confirm successful loading of Bi_2_O_3_ on bentonite. In addition, it is observed disappearance of the peak at 6.5° which is explained due to loss of water between the clay layers through calcination process^53^. In Fig. 11c, a decrease of intensity of broad peaks related to amorphous nature of unsaturated polyester at 19.33°, 28.74°, 40.64° is observed^7,54^. Also, it is observed the appearance of sharp peaks at 11.27°, 23.29°, 26.02°, 28.59°, and 49.63° which is related to added nanoparticles. The presence of these sharp peaks reflects increasing of composite’s crystallinity with addition of nanoparticles^9,16^.

### Thermal gravimetric analysis (TGA)

**Fig. 12:**
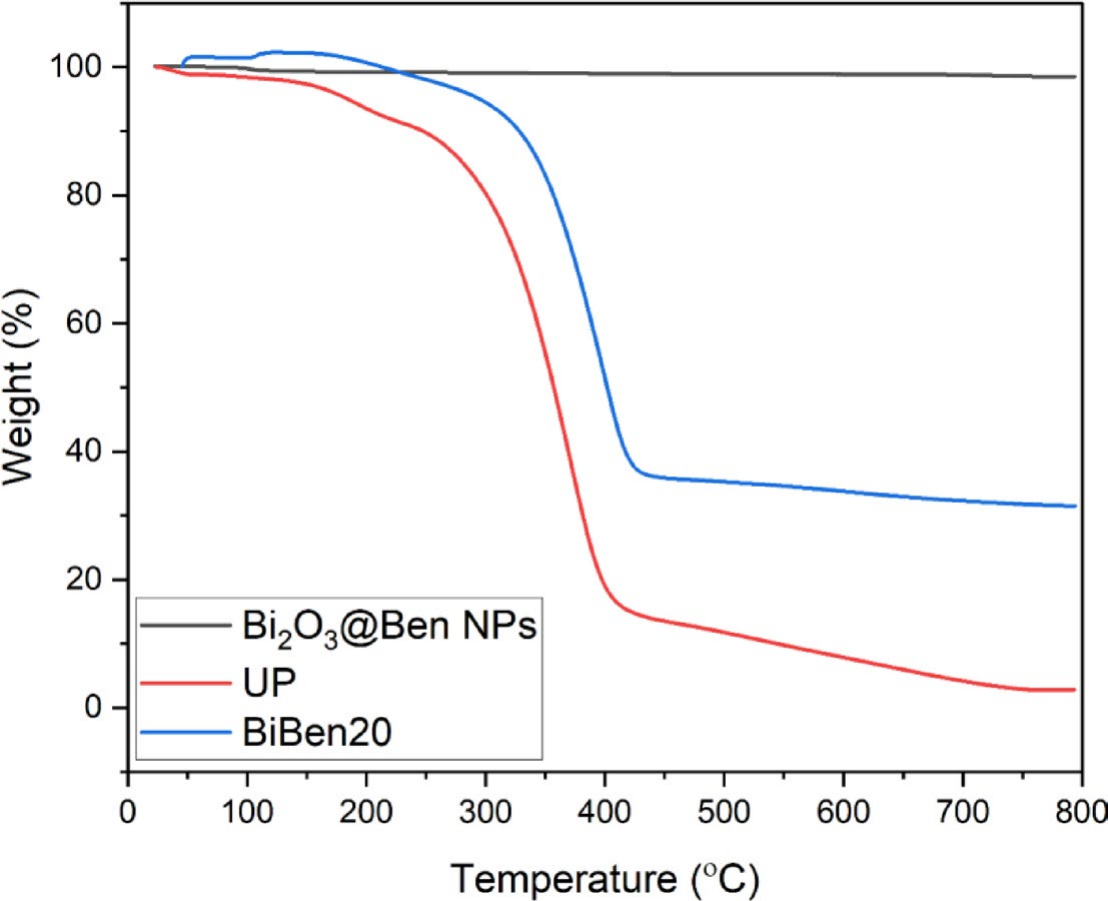
TGA curve of bentonite supported Bi_2_O_3_ NPs, pure UP, and BiBen20 nanocomposite.

Figure 12 Shows the TGA curve of bentonite supported Bi_2_O_3_ NPs, pure UP, and 20 wt% nanocomposite (BiBen20). The prepared nanoparticles are seen to exhibit a high degree of thermal stability up to 800 °C, at which point they lost roughly 1.52% of their weight. The evaporation of adsorbed water on the surface of synthesized nanoparticles could be the reason for this very slight weight reduction^10,31^. Additionally, it can be observed that pure UP lost 50% of its weight at 357 °C and 10% at 247 °C. However, the 20 wt% nanocomposite broke down at higher temperatures, losing 10% of its weight at 328 °C and 50% at 402 °C. This suggests that adding bentonite supported Bi_2_O_3_ NPs slows down the thermal degradation of unsaturated polyester and increases its thermal stability^4^.

### Scanning electron microscope

**Fig. 13:**
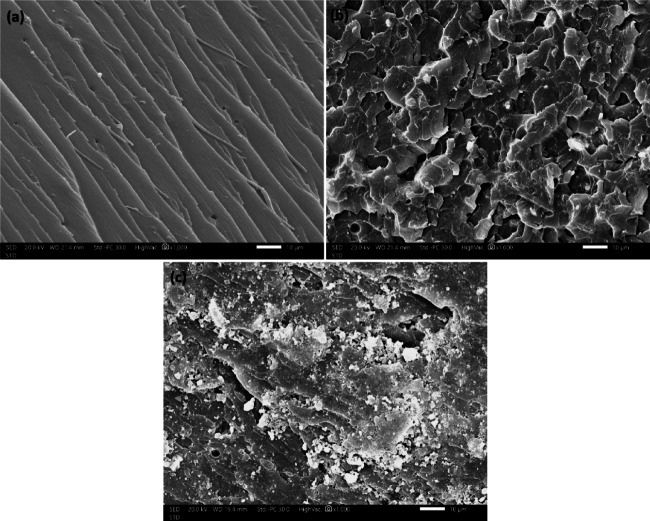
SEM images of (**a**) pure UP, (**b**) BiBen5 nanocomposite, and (**c**) BiBen20 nanocomposite.

Figure 13 Shows SEM images of fracture surfaces of pure UP, 5 wt% nanocomposite (BiBen5), and 20 wt% nanocomposite (BiBen20). The smooth fracture surface for pure UP in Fig. 13a indicates the brittle fracture of unsaturated polyester^4,48^. On the other hand, the nanocomposites surface (Fig. 13b and c) has flakes like structure due to presence of bentonite. Also, it is observed that Bi_2_O_3_ particles are dispersed on bentonite surface with presence of some agglomeration at higher filler percent.

### Compressive strength test

**Fig. 14:**
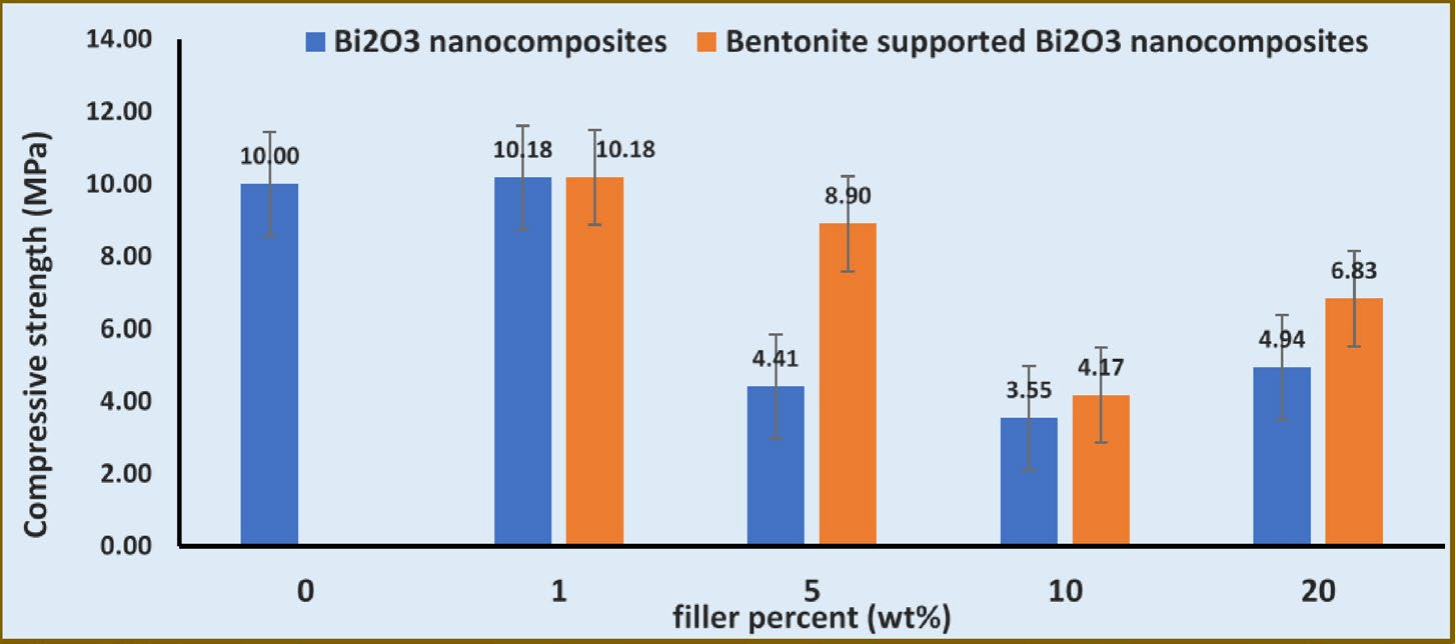
Compressive strength for the nanocomposites containing different percentages of Bi_2_O_3_ NPs and bentonite supported Bi_2_O_3_ NPs.

The mechanical properties of nanocomposites were evaluated through compressive strength test. Figure 14 Shows values of compressive strength for UP and nanocomposites with different percentages of Bi_2_O_3_ NPs and bentonite supported Bi_2_O_3_ NPs. The 1 wt% nanocomposite shows a small increase in compressive strength compared to pure UP, which could be the result of the nanoparticles’ good dispersion in the composite. The compressive strength then drops as the ratio of nanoparticles rises, which could be caused by nanoparticles agglomeration and their adverse effect on the crosslinking reaction during the composite’s curing process^4,22^. To evaluate the effect of bentonite addition on mechanical properties of the nanocomposites, the compressive strength values of bentonite supported Bi_2_O_3_ nanocomposites are compared with the values of Bi_2_O_3_ nanocomposites from the previous work^21^. It is observed that bentonite supported Bi_2_O_3_ nanocomposites have higher compressive strength than Bi_2_O_3_ nanocomposites at the same filler percent which indicates that addition of bentonite enhances mechanical properties of composites and moderates negative effect of Bi_2_O_3_ reported in previous study.

### Radiation attenuation properties

**Fig. 15:**
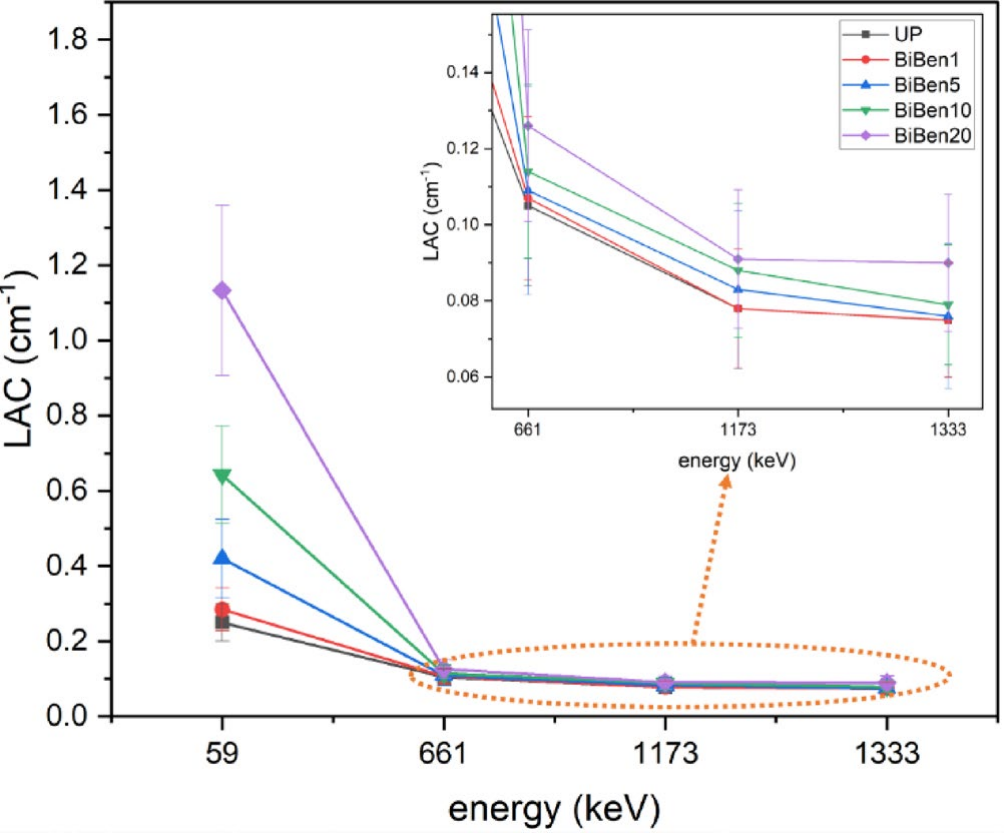
experimental LAC for UP and different composites at different energies.

LAC values for unsaturated polyester and other composites at various energies are displayed in Fig. 15. It is found that LAC has greater values at lower energies (59 keV) and diminishes with increasing energy. This is attributed to the fact that the photoelectric effect is more common at lower energies, but Compton scattering and pair creation are more common at higher energies. Additionally, it is shown that the LAC increases as the added nanoparticles ratio increases which can be attributed to the increase in density of composites where LAC is directly proportional to density. For example, it increases significantly from 0.250 cm^-1^ for pure unsaturated polyester to 1.133 cm^-1^ for 20 wt% nanocomposite at 59 keV. On the other hand, it increases somewhat at higher energies because the photoelectric effect dominates at lower energies^55,56^.

Table 2 shows the experimental and theoretical values of LAC and the relative deviation between them where the relative deviation was calculated according to the following equation:

**Table 2:** values of experimental, theoretical LAC and RD% between them.

	59 keV			661 keV			1173 keV			1333 keV		
	Exp	XCOM	RD%	Exp	XCOM	RD%	Exp	XCOM	RD%	Exp	XCOM	RD%
UP	0.250	0.247	−0.87	0.105	0.107	2.02	0.078	0.082	4.60	0.075	0.076	2.02
BiBen1	0.285	0.283	−0.75	0.107	0.107	−0.66	0.078	0.081	3.31	0.075	0.076	0.84
BiBen5	0.420	0.443	5.41	0.109	0.112	2.71	0.083	0.083	0.11	0.076	0.078	3.32
BiBen10	0.643	0.663	3.00	0.114	0.118	3.08	0.088	0.088	0.29	0.079	0.082	4.05
BiBen20	1.133	1.122	−0.94	0.126	0.128	1.25	0.091	0.093	2.24	0.090	0.087	−3.68

$$RD\%=\frac{LAC\left(XCOM\right)-LAC(exp.)}{LAC(exp.)}*100$$

It is noticed that relative deviation for all samples doesn’t exceed 6%, which indicates compatibility between experimental and theoretical results.

**Fig. 16:**
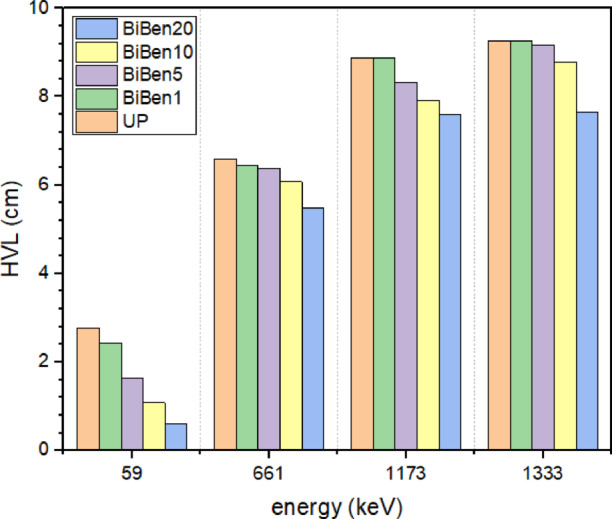
HVL values of UP and different composites at different energies.

**Fig. 17:**
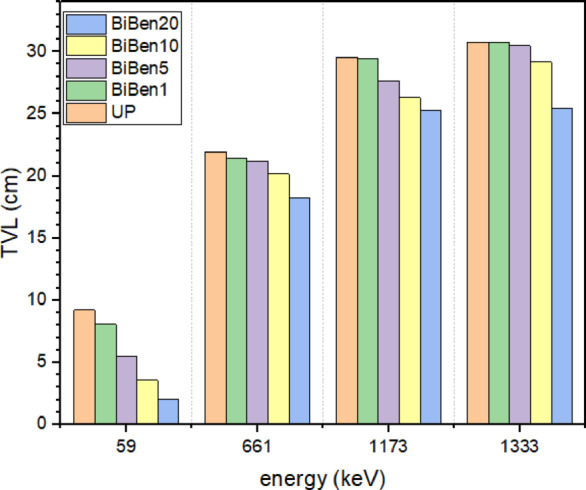
TVL values of UP and different composites at different energies.

**Fig. 18:**
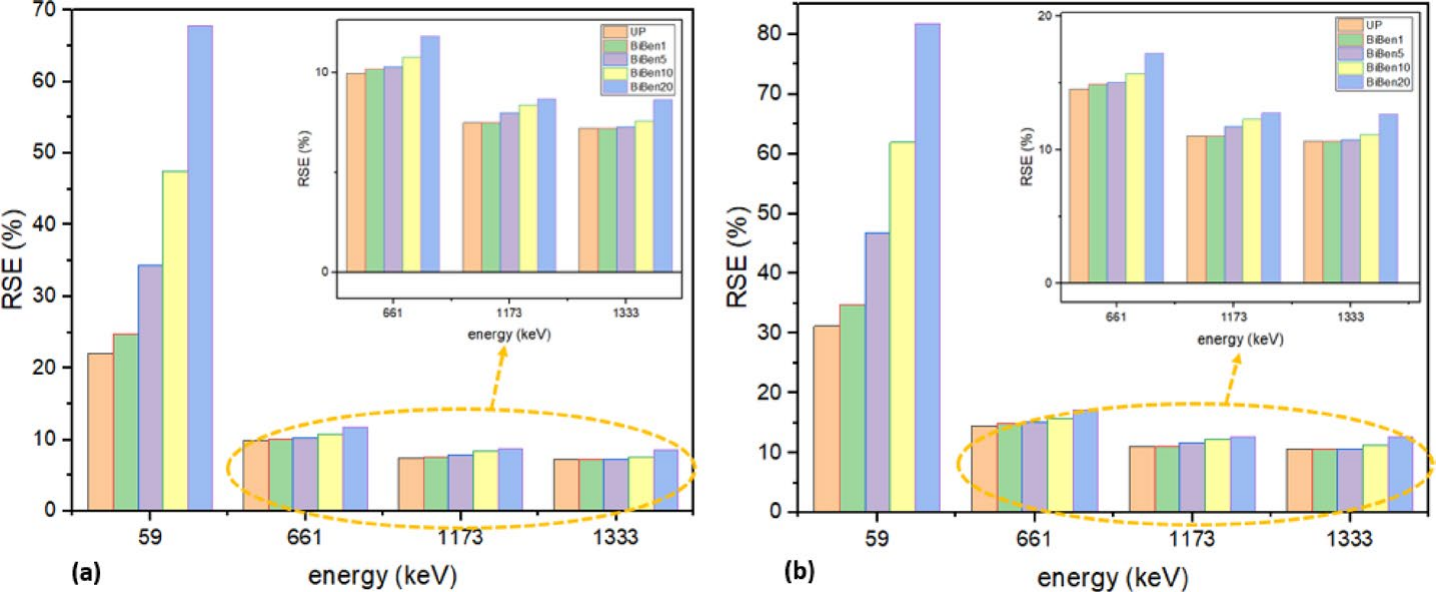
RSE% of UP and different composites at (**a**) 1 cm and (**b**) 1.5 cm thickness.

As predicted, Fig. 16 illustrates how HVL values rise with energy, where HVL is inversely proportional to LAC and more thickness is needed to stop higher energy photons from penetrating. Additionally, it demonstrates how adding 20 wt% of the prepared nanoparticles enhances HVL values, with unsaturated polyester’s HVL value improving from 2.77 to 0.61 cm at 59 keV. Because the photoelectric effect predominates at low energies, a thin layer is sufficient to absorb half of photons. However, at 1333 keV, when the photoelectric effect becomes less important and Compton scattering and pairs production become more significant at higher energies, a similar addition percent slightly reduces the HVL value from 9.25 to 7.66 cm where the photoelectric effect is greatly influenced by atomic number (photoelectric effect ∝ Z^4–5^). So, increasing filler loading leads to rapid increase of attenuation capability at low energies while Compton scattering and pair production are less affected by atomic number (Compton scattering ∝ Z and pair production ∝ Z^2^) which may explain why attenuation properties slightly improve with increasing filler content at higher energies^57,58^. TVL values follow the same patterns as HVL values (Fig. 17). Additionally, as more thickness is required to reduce radiation intensity to a tenth of its value, TVL values are found to be higher than HVL values. The TVL value of unsaturated polyester increases from 9.22 to 2.03 cm at 59 keV and from 30.74 to 25.45 cm at 1333 keV when 20 wt% of the nanoparticles are added. This suggests that the low thickness of 20 wt% nanocomposites is helpful in attenuating low energy photons. Radiation shielding efficiency is another parameter that was measured. Shielding efficiency at various energies for varying thicknesses is displayed on Fig. 18 It demonstrates a reduction in shielding efficiency as photon energy increases, which is explained by the photoelectric effect’s dominant role at lower energies in the previously described parameters. Additionally, it is found that the 20 wt% nanocomposite has the best shielding efficiency at 59 keV (67.79% and 81.72% at 1 and 1.5 cm thickness, respectively) compared to pure unsaturated polyester (22.09% and 31.24% at 1 and 1.5 cm thickness, respectively), demonstrating its strong ability to attenuate low energy photons. Although increasing filler content increases attenuation capability of composites, it leads to decrease in mechanical properties. However, it doesn’t limit using of these composites where it can be fabricated with suitable filler loading that doesn’t negatively affect its performance according to desired application and energy of attenuated radiation. These nanocomposites may be particularly useful for practical shielding applications such as the lightweight protective panels found in diagnostic X-ray rooms, dental radiography offices, and mammography centers. Also, it can be utilized to create building materials for radiation-safe walls and partitions.

**Fig. 19:**
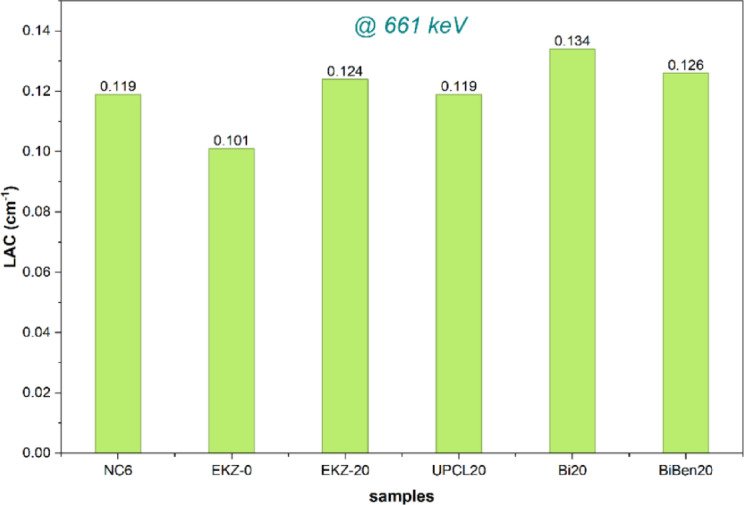
comparison between LAC values of different composites at 661 keV.

When LAC values of different composites including present and previous works (BiBen20 (present work), unsaturated polyester containing 20% Bi_2_O_3_ NPs (Bi20)^21^, unsaturated polyester containing 5% nano-clay and 20% PbO (UPCL20)^4^, epoxy filled with 10% kaolin clay (EKZ-0)^59^, epoxy filled with 20% kaolin clay and 20% ZnO (EKZ-20)^59^, and polypropylene containing 40% nano-bentonite (NC6)^31^) are compared (Fig. 19), it is observed that present results are in agreement with previous studies at close filler loading and the same energy. Furthermore, the difference between LAC values of Bi20 and BiBen20 is about 6% which reflects good performance of our composite as shielding materials in addition to positive effect of bentonite on compressive strength.

## Conclusion

In this study, unsaturated polyester was reinforced with different amounts of bentonite supported Bi_2_O_3_ NPs (1, 5, 10, and 20 wt%) and the shielding properties of these composites were studied at different energies (59, 661, 1173, and 1333 keV). The formation of Bi_2_O_3_ NPs on bentonite surface was done by green synthesis method using olive leaves extract. The TEM images showed successful loading Bi_2_O_3_ NPs on bentonite surface. Also, it is confirmed by EDX analysis, IR, and XRD. The results showed decrease in LAC and RSE% values and increase in HVL and TVL values with increase in energy. Also, it was observed that attenuation properties were improved with increase of filler ratio where the 20 wt% composite sample has the highest LAC values. This addition ratio also improved thermal properties and retarded the thermal degradation of nanocomposites. In terms of mechanical properties, the presence of bentonite slightly enhanced the compressive strength of composites. However, increasing filler content causes a decrease in compressive strength. So, future studies are recommended to focus on improving mechanical properties of higher filler loading composites.

## Data Availability

The data presented in this study are available on request from the corresponding author.
